# Employee Health and Wellness Outcomes Associated With Perceived Discrimination in Academic Medicine: A Qualitative Analysis

**DOI:** 10.1001/jamanetworkopen.2021.45243

**Published:** 2022-01-28

**Authors:** Matthew D. Kearney, Frances K. Barg, Dominique Alexis, Eve Higginbotham, Jaya Aysola

**Affiliations:** 1 Department of Family Medicine and Community Health and Research, Perelman School of Medicine, University of Pennsylvania, Philadelphia; 2 Center for Health Equity Advancement, Perelman School of Medicine, University of Pennsylvania, Philadelphia; 3 Office of Inclusion and Diversity, Perelman School of Medicine, University of Pennsylvania, Philadelphia; 4 Leonard Davis Institute of Health Economics, University of Pennsylvania, Philadelphia; 5 Penn Medicine Center for Health Advancement, Perelman School of Medicine, University of Pennsylvania, Philadelphia

## Abstract

**Importance:**

Organizational culture and workplace interactions may enhance or adversely impact the wellness of all members of learning and work environments, yet a nuanced understanding of how such experiences within health care organizations impact the health and wellness of their membership is lacking.

**Objective:**

To identify and characterize the reported health and wellness outcomes associated with perceived discrimination among academic medicine faculty, staff, and students.

**Design, Setting, and Participants:**

This qualitative study analyzed anonymously submitted written narratives from 2016 that described experiences related to inclusion in the workplace or lack thereof. Narratives that described health outcomes associated with work- or school-based discrimination were purposively sampled. Participants were faculty, staff, and students at health-related schools or hospitals affiliated with the University of Pennsylvania. Data analysis was performed from March 2019 to January 2020.

**Exposures:**

Self-reported experiences, both witnessed and personal, of discrimination in the workplace.

**Results:**

A total of 315 narratives were collected, and 115 narratives from 115 participants were analyzed. Most respondents identified as female (70 respondents [60.9%]), non-Hispanic White (68 respondents [59.1%]), and heterosexual (89 respondents [77.4%]) and had worked at the institution for at least 1 year (99 respondents [86.0%]). The outcomes associated with adverse workplace experiences were broad and ranged in nature from emotional to mental and physical. Most reported outcomes were emotional (101 respondents [87.8%]), and more than 1 in 10 narratives (14 respondents [12.2%]) described a mental or physical health outcome. Many of the participants felt devaluated, overexerted, and hopeless, resulting in clinically relevant manifestations, such as increased stress and anxiety levels and even elevated blood pressure.

**Conclusions and Relevance:**

This qualitative study identified a continuum of negative outcomes on employee health and well-being associated with perceived discrimination and chronic exclusion in the workplace. These findings suggest the need for organizations to promote inclusion as a component of workplace wellness interventions.

## Key Points

**Question:**

What are the reported associations of workplace exclusion and perceived discrimination with employee health behaviors and outcomes in a large academic health care institution?

**Findings:**

In this qualitative study of 115 participants, most reported outcomes associated with perceived discrimination were emotional (87.8%). More than 1 in 10 narratives (12.2%) described a mental or physical health condition, including stress, depression and anxiety, posttraumatic stress disorder, changes in blood pressure, and changes in sleeping behaviors.

**Meaning:**

These findings suggest that creating diverse and inclusive environments in which to work and learn can play a vital role in reducing harms associated with perceived discrimination and exclusion.

## Teaser

This qualitative study aims to identify and characterize the reported health and well-being outcomes associated with perceived discrimination among academic medicine faculty, staff, and students.

## Introduction

Inclusion in the workplace, or a lack thereof, is associated with workforce mental and physical wellness and has implications for public health interventions. Even among diverse workforces, workplace dynamics and organizational culture can create exclusive environments where some employees continue to experience perceived discrimination (PD).^1^ PD has harmful associations with employee health behaviors and health outcomes.^2,3,4^ In 2020, 1 in 2 workers in the US reported feeling discriminated against by a colleague.^5^ Health care workforces are unique cross-sections of the general population who often experience occupational stress resulting from workplace expectations, time constraints, and lack of social support,^6^ and may offer a unique perspective as both health care practitioners professionally and health care consumers personally. Within health care settings, the total workforce is also diverse and is composed, accordingly, of students and trainees, educators and faculty, and both clinical and nonclinical employees. Given the importance of fostering inclusive environments in academic medical settings and recruiting and retaining a diverse body of employees and trainees, it is critical to understand how a lack of inclusion contributes to the health and wellness of underrepresented or historically marginalized populations by exploring their experiences of PD and exclusion.

PD includes exclusion, bullying, harassment, microaggressions, or even violence.^7,8,9^ Previous survey research has identified associations between the impacts and perceptions of workplace discrimination in health care settings among nurses,^10^ practicing physicians,^11^ and entire health care institutions.^12^ Yet to the best of our knowledge, we lack qualitative assessments of experiences of such behaviors across entire health care organizations to characterize the range or extent of outcomes on members of this environment, including their coping mechanisms and health impacts. Such assessments could help inform organizational efforts to reduce the prevalence and impact of exclusion and discrimination. Therefore, to address this identified gap in the literature and contribute qualitative evidence on the impact of perceived workplace discrimination in an academic health care setting we examined narratives from health care employees and trainees to identify and characterize the reported outcomes associated with workplace exclusion and discrimination on employee health behaviors and wellness.

## Methods

### Setting, Design, and Study Population

We conducted a qualitative analysis of written narratives focused on the reported impact of PD and a lack of workplace inclusion. Respondents were employees, students, and trainees of participating hospitals and schools from a large academic health care institution in Philadelphia, Pennsylvania. Ethical approval was obtained by the University of Pennsylvania institutional review board. This study adhered to the Standards for Reporting Qualitative Research (SRQR) reporting guideline. Participants provided informed consent through the survey.

### Data Collection and Sampling

We analyzed qualitative data collected by members of the study team (M.D.K., F.K.B., E.H., and J.A.) between June 6 and July 8, 2016, from an online call-for-narratives campaign titled *Please Tell Me Your Story.* All analysis reported in the current study was completed between March 2019 and January 2020. Methodological details and findings from the prior analysis are reported elsewhere.^13^ Participants were recruited via email to anonymously participate in the online call-for-narratives, which was administered through REDCap (version 2016; Vanderbilt University). The call-for-narratives consisted of 2 open-ended prompts. The first open-ended prompt asked about participants’ specific experiences with inclusion or lack thereof; the second open-ended prompt asked about the general climate surrounding inclusion at the University of Pennsylvania. Narratives were eligible for further analysis if respondents answered both prompts and provided demographic information. Narratives with completed respondent demographic variables were collected, and the content was analyzed to identify factors associated with workplace inclusion, including whether the narrative described workplace discrimination or its impact.^13^ Race and ethnicity were self-reported by the respondents and were assessed in this study because we wanted to examine differences in experiences with inclusion between different populations of respondents.

Of the narratives in the complete data set, those describing the effects of discrimination on the narrator were purposively selected for further analysis in the current study to understand the health outcomes of discrimination. Respondent demographic characteristics for narratives in this sample did not differ significantly from respondents in the full sample.

### Statistical Analysis

Informed by prior research into workplace characteristics and employee burnout,^12,14,15^ we developed a codebook to characterize the reported impacts of workplace discrimination among the sample of relevant narratives. Outcomes associated with PD were initially coded as emotional, mental, or physical. Emotional outcomes were defined as cognitive, emotional, and social responses to external stimuli (ie, environmental, interpersonal). Narratives coded as physical or mental outcomes included those that would be typically clinically diagnosed according to available *International Statistical Classification of Diseases and Related Health Problems, Tenth Revision *diagnostic codes (eg, posttraumatic stress disorder [PTSD]),^16^ so the code was renamed “mental health and physical effects” for parsimony. Although many of the identified emotional outcomes may be characterized as symptoms or precursors of mental and physical health conditions, we coded these separately. The final codebook dichotomized effects reported in narratives as either emotional or mental and physical outcomes. The initial codebook was developed by 4 members of the study team (M.D.K., FK.B., J.A., and E.H.) and was later refined by 2 members of the team (M.D.K. and J.A.). Modifications to the codebook were informed by group discussions about emerging patterns observed in narratives. All narratives were then coded by 2 members of the study team (M.D.K. and D.A.), and discrepancies were resolved through achieving group consensus during in-person team meetings. Coding was facilitated by NVivo software version 11 (QSR International).

## Results

### Sample Characteristics

Of the 315 narratives in the complete data set, 115 (36.5%) described the impact of discrimination on the narrator. As shown in Table 1, most respondents identified as female (70 respondents [60.9%]), non-Hispanic White (68 respondents [59.1%]), and heterosexual (89 respondents [77.4%]). Nearly 4 in 10 (43 respondents [37.5%]) were people of color. Thirteen participants (11.3%) identified as lesbian, gay, or bisexual. Most respondents had worked at the institution for at least 1 year (99 respondents [86.0%]). Staff was the most frequent position held (46 respondents [40.0%]).

**Table 1.** Respondent Sociodemographic Characteristics

Characteristic	Respondents, No. (%) (N = 115)
Gender identity	
Female	70 (60.9)
Male	38 (33.0)
Other^a^	4 (3.5)
Decline to answer	3 (2.6)
Race or ethnicity	
Asian	16 (13.9)
Hispanic or Latino	4 (3.5)
Non-Hispanic Black	9 (7.8)
Non-Hispanic White	68 (59.1)
Other^b^	14 (12.2)
Decline to answer	4 (3.5)
Religion	
Judaism or Christianity	61 (53.1)
None	29 (25.2)
Other^c^	20 (17.4)
Decline to answer	5 (4.4)
Sexual orientation	
Straight or heterosexual	89 (77.4)
Lesbian, gay, or bisexual	13 (11.3)
Decline to answer	11 (9.6)
Other^d^	2 (1.7)
Disability status	
No	96 (83.5)
Decline to answer	15 (13.1)
Yes	4 (3.5)
Language	
English	102 (88.7)
Other^e^	9 (7.8)
Decline to answer	4 (3.5)
Position	
Staff	46 (40.0)
Faculty	33 (28.7)
Graduate student or postdoctoral fellow	18 (15.7)
Trainee (resident, fellow, or intern)	14 (12.2)
Undergraduate student	3 (2.6)
Decline to answer	1 (0.9)
Time at institution, y	
>10	33 (28.7)
≥2 to <5	24 (20.9)
≥5 to <10	24 (20.9)
<1	16 (13.9)
≥1 to <2	15 (13.0)
Decline to answer	3 (2.6)
Primary area	
School of Medicine	51 (44.4)
Hospital	45 (39.1)
School of Nursing	6 (5.2)
Other	6 (5.2)
School of Dental Medicine	4 (3.5)
Decline to answer	3 (2.6)

^a^ Includes transgender, gender nonconforming or gender queer (ie, does not identify as male or female), and “other.”

^b^ Includes Native American or Alaskan Native, Pacific Islander, and “other.”

^c^ Includes Muslim, Buddhist, Unitarian-Universalist, Hindu, Native American, Sikh, and “other.”

^d^ The survey choice was “other.” Respondents were not asked to specify.

^e^ Includes Mandarin, Hindustani, Spanish, Russian, Arabic, Bengali, Portuguese, French, and “other.”

### Self-reported Outcomes Associated With PD

PD emerged saliently: of the 315 original narratives, 115 described outcomes associated with PD. Most outcomes (101 respondents [87.8%]) were emotional and more than 1 in 10 narratives (14 respondents [12.2%]) described a mental or physical health condition. Nearly one-third of narratives (35 respondents [30.4%]) described events witnessed rather than personally experienced by participants. To decontextualize responses, unique identifiers (eg, participant X) are used in the body of the below text. Table 2 presents sociodemographic characteristics for each alias.

**Table 2.** Respondent Aliases and Select Demographic Characteristics

Participant	Gender identity	Race or ethnicity	Time at institution, y	Position
A	Female	White	≥10	Faculty
B	Male	White	6-10	Staff
C	Female	Black	1-5	Postdoctoral fellow
D	Male	White	≥10	Staff
E	Female	White	1-5	Staff
F	Male	White	≥10	Faculty
G	Female	White	≥10	Staff
H	Other	Asian	1-5	Staff
I	Male	Asian	≥10	Postdoctoral fellow
J	Female	White	1-5	Graduate student
K	Male	Hispanic or Latino	1-5	Postdoctoral fellow
L	Female	White	<1	Staff
M	Female	White	1-5	Undergraduate student
O	Female	Black	6-10	Staff
P	Female	White	≥10	Staff
Q	Male	Asian	1-5	Postdoctoral fellow

### Outcomes on Emotional Well-being

As shown in Table 3, respondents described various emotional outcomes associated with PD. These included hopelessness, devaluation and disrespect, discomfort and intimidation, and isolation.

**Table 3.** Narrative Examples of Emotional Outcomes Associated With Perceived Discrimination

Emotional outcome	Representative quotation
Feelings of hopelessness	“There are many respectful people [at the University]. What is…lacking is a robust mechanism to appropriately deal with those who are disrespectful. It should not be acceptable for administrators, physicians, and others to lie in their dealings with faculty.” Participant A
	“My supervisor, while being careful not to cross any discriminatory lines, continually referred to certain of his/her employees as stupid, disloyal, etc. There was no respect afforded to employees in general. Everything had to revolve around the supervisor. He/she was extremely self-centered. He/she has been written up a number of times, but since this person has tenure nothing has been done, and I am sure nothing will be done.” Participant B
Feelings of devaluation and disrespect	“There is no particular incident that stands out in my mind as a positive or negative inclusion experience. I feel unwelcome/unvalued subtly on a daily basis, however. I am an African American resident, but there are no other black residents in my ENTIRE program…I feel unvalued as a minority. I also feel like my career would not prosper at [the University] as well as other comparable northeastern programs due to microaggressions leading to an inability to have my work recognized and my career promoted appropriately. I do not intend to stay at [the University] after my residency training for these reasons…. As a whole, [the University] is a high achieving institution without snobbery.” Participant C
	“I have witnessed a few instances where women or non-white students or employees were treated in a disrespectful or discriminatory manner, and I conclude that, despite the rules and policies, it all depends on the particular individuals you end up dealing with…. In the somewhat limited sector that I have contact with, I would consider the atmosphere to be inclusive and respectful—but that is from the perspective of a white male observer, not a likely victim.” Participant D
Feelings of discomfort and intimidation	“The senior male faculty within our department are disrespectful to the woman staff members. On several occasions I have felt bullied by the male faculty to process transactions outside the financial policy. The most recent example happened during the annual salary increase process. One of our faculty members bullied and pressured me into submitting salary increases for our department that do not average to 3% per policy.… I felt very intimidated by his words and body language.” Participant E
	“Unfortunately the academic environment allows strong-willed abusive personalities to rise to positions of management without adequate checks and balances on behavior. For a dozen years I have been in a hostile work environment characterized by verbal and on a few occasions physical abuse of other physicians, staff and patients…. Consequences include a 90% drop in academic productivity, ZERO promotions on the CE track in more than 10 years, and a dozen faculty quitting over this time period. Serial chairman have turned a blind eye to this behavior. The culture of intimidation is such that individual physicians and staff will not report incidents for fear of reprisal and jeopardizing their careers and the expectation that nothing will change. This negative culture transcends race and gender. [The University] needs to educate and strengthen its leaders to change this culture…. Over the past 20 years I have seen our training pool become ever more diverse, with white males now in the minority. The atmosphere in my section is quite neutral to race, ethnic origin, and gender.” Participant F
	“There was a person in a position of power in the department who told me she enjoyed playing ‘good cop bad cop’ to keep employees in their place and pit them against each other so that they would only trust her. She was under the impression that this was the best way to get employees to do good work? I knew this was the way she operated, but I could not believe she actually came out and told me. She seemed very proud of the way she manipulated people (including her superiors).” Participant G
Feelings of isolation	“A woman knew I was Asian and kept making Asian jokes. She had a close relationship with the office manager and I didn’t feel I could say anything. She worked next to me and my other friends in the office invited her to lunch so I started wearing noise canceling headphones and eating alone…. I generally find the climate good. I loved walking into the main lobby of the university hospital to see celebrations of Hanukkah, Kwanzaa, and Christmas.” Participant H
	“I was not welcomed by my lab mates when I first joined the lab. They ignored me a lot and often treated me in undignified ways. My PI was completely ignorant/oblivious of the situation and his preference to socialize only with local Americans in the lab didn’t help with the situation…. There isn’t much inclusion at all especially among researchers. People only communicate with each other for research needs and minimally for socializing. Exclusion happens even among peers in the lab simply due to cultural exclusion or prior cultural assumptions made. I would have quit the lab and joined another if it wasn’t for the visa restrictions.” Participant I
	“As a female graduate student, I would be told to participate more in lab meetings and come up with experiment ideas. But the male PI, and his two male postdocs would often meet informally in his office in the evening drinking and smoking while they came up with ideas and I was not invited (nor did I feel comfortable attending).” Participant J

Abbreviations: CE, clinician educator; PI, principal investigator.

#### Feelings of Hopelessness

Participant narratives described different factors that were associated with feelings of hopelessness. Some respondents discussed a lack of recourse for reporting workplace behaviors, either as a general comment on current practices, or on the basis of their personal experiences. Participant A, a senior female faculty member, felt generally positive about her colleagues, but still expressed a desire for better reporting practices (ie, improved accountability, expanded authority). Power dynamics between tenured and nontenured (or non–tenure-track) participants also contributed to some participants’ hopelessness, as discussed by participant B. Participants A and B also demonstrated another noteworthy characteristic of narratives: sandwiching concerns between positive comments or disclaimers of exceptionality to provide a cognitive frame through which the reader should interpret the narrator’s perspective. Such framing by the narrators may suggest a belief that PD and its sequelae are the result of 1 or more person’s conduct and not a reflection of overall workplace culture.

#### Feelings of Devaluation and Disrespect

Respondents commonly felt that their colleagues looked down on them or other persons because of their racial or ethnic backgrounds and that these perceptions may have had an impact on their mental health through increased feelings of devaluation. Participant C felt unvalued and unwelcome as a result of frequent microaggressions, associating these daily occurrences with overall issues in workforce diversity, motivating them to seek employment elsewhere. Microaggressions are typically casual comments or questions with underlying contexts of bias and discrimination, such as describing oneself as color blind (“I don’t see a person’s race”) or ascriptions of intelligence (“You are so articulate!”). Although participant D did not discuss any personal experiences of discrimination, his narrative mentioned several instances of discrimination and disrespect against “women and non-white students or employees.”

#### Feelings of Discomfort and Intimidation

Workplace interactions were reported that caused participants or recipients of abuse in their narratives to experience discomfort and intimidation. Participant E, a female respondent, discussed intimidation by a male colleague, stating that their narrative shared 1 of several experiences where male colleagues, both supervisors and subordinates, had used words or body language that affected them negatively. Although personal narratives that expressed discomfort or intimation were typically authored by female respondents who described male aggressors, there were exceptions. For example, participant F, a veteran male senior faculty, wrote about a culture of intimidation not attributable to a single person, while pointedly stating that intimidation was universal (ie, nondiscriminatory). In participant G’s department, a female supervisor abused her position of power by manipulating other employees without receiving consequences.

#### Feelings of Isolation

Whether through exclusion or reclusion, social isolation was a powerful result of PD, and narrators frequently discussed its impact on their self-worth, dignity, and respect. Participants described how they felt their colleagues perceived them because of demographic or other characteristics. Several believed that colleagues even excluded them from work-related situations because of these characteristics. Participant H was offended by their colleagues’ racial stereotype jokes and reacted by eating alone and wearing noise-canceling headphones, a unilateral solution motivated both by participant H’s perception of workplace power dynamics and hopelessness. Participant I felt that his colleagues’ ostracizing behavior was driven by his supervisor’s xenophobic attitude and felt hopeless to improve his situation because of visa requirements. Finally, participant J was frustrated by their colleagues’ gender-based exclusion and simultaneous demands for better collaboration, echoing participant I’s narrative of how colleagues form cliques along demographic lines.

### Mental and Physical Health Outcomes

The health outcomes identified in this section are known to be associated with mental and physical health, many of which are clinically diagnosable (Table 4). In some cases, narrators state how their experiences cause them to prioritize their workplace responsibilities above all else, at the expense of their health and physical well-being. The outcomes characterized include stress, depression and anxiety, PTSD, changes in blood pressure, and changes in sleeping behaviors.

**Table 4.** Narrative Examples of Mental and Physical Health Outcomes Associated With Perceived Discrimination

Health outcome	Representative quotation
Stress	“A colleague of mine is currently experiencing exclusion and devaluation…. The aforementioned colleague is the last remaining employee that served under the original supervisor, the most experienced and competent employee at the core, and a member of an underrepresented minority. Yet, this person’s job is in jeopardy and they face daily harassment from a supervisor that wishes them to leave or be dismissed for no decent or fair reason. To compound matters, my colleague’s attempts to go above the supervisor have been met with dismissal and the general sentiment that the issue should not be the supervisor’s boss’s concern. The colleague is currently suffering the stress and humiliation of having their ability and standard of work officially reviewed after years of providing exemplary services to a money-making and money-saving university facility. Their job is at risk and, no matter the outcome of the review, the best they can hope for is to return to a decidedly hostile work environment and face further torment from the supervisor.” Participant K
Depression and anxiety	“In my first few weeks of working here, it was clear that there is an underlying negative, overly critical judgmental environment in the department I work for…. First of all, it made me extremely fearful and anxious in my position of making even the most minor mistake and also, what will be said of me by others when I was out of earshot. Further, it leads to others having an overall negative opinion of the other person, and isn’t constructive to a positive, problem solving environment…. It is pervasive…. In all of my years of working, I have never been part of an environment where everyone seems to be so fearful of making mistakes. I’m sad that is has been my experience at [the University], and I hope it will improve, but in the meantime, I’m looking for other opportunities.” Participant L
	“I was working in my thesis lab. Things were good, tough, but good…. Then my mom died unexpectedly. My PI told me take all the time I needed…. At the end of the semester I told him I needed to be home for the full holiday break to evaluate if I needed to take a leave of absence. He agreed but was clearly not happy…. I came back and immediately did not feel welcome…. I changed labs but had a very hard time finding one with funding, despite having my own grant. I finally did find one, finished my prelim, but was ultimately derailed by the whole experience. I’ve been dealing with severe depression and anxiety and have been on a leave of absence since.” Participant M
Posttraumatic stress disorder	“A disabled, minority research assistant endured systematic bullying within her department for approximately 2 years. While working under the director that hired her, she was a flourishing and very productive member of the department (the director called her a ‘workhorse’). However, once a new director started (who was promoted internally into the position) work life changed…. Over the next year and a half, the assistant reported the bullying to HR as well as her director’s immediate boss—to no avail. She told she was being ‘too sensitive’ when her boss made comments about her weight (gain due to meds as well as stress from bullying), her hair (director = Caucasian, assistant = African-American, the assistant was losing her hair due to illness and was wearing extensions as a result)…. Over time, the once bubbly assistant became (what seemed to be) clinically depressed, and so fearful of the director that she literally froze in place whenever she passed by her cubicle. After looking for nearly a year, she secured a new position at [the University], and left the department…. She is currently being treated for PTSD from the experience, while in her new position.” Participant N
Changes in blood pressure	“There was a person in a position of power in [a clinical role] who told me they enjoyed playing ‘good cop bad cop’ to keep employees in their place and pit them against each other so that they would only trust her. She was under the impression that this was the best way to get employees to do good work?…It was a very toxic work environment. I left for another job because it was actually affecting my health working in such an unhealthy drama filled environment. I gained 15 pounds while working in [the department] and my blood pressure shot up, and I have always had low blood pressure all my life. I felt really helpless because even though I was a University employee, this person and most of the people in the department are [hospital, not University] employees, so I didn’t even know who to turn to for help.” Participant P
Changes in sleep behaviors	“Many internationals compromise personal dignity and values so that they don’t get kicked out, but ultimately it only promotes intolerance and behavior aimed at silencing their voices and opinions rather than the making them feel included and welcome…. I devoted my full effort in understanding the ‘large’ lab culture and tried my best to navigate my way through the thickets of largely unwelcoming and unfriendly environment for newcomers. I did succeed in settling in solely due to efforts and ability to keep my head down and also to large extent by spending countless hours/week (around 12-14/h) in the lab. I traveled back home to NC every other weekend and many times took long weekends (Monday off) to be able to drive back and also to be able to spend enough time with family back home and for doctors visits etc. Although, I did miss the second and third ultrasound and the live news that we were expecting a baby girl, anyways…on many many Mondays or on Fridays when I drove I was too tired and should not have been driving 500 miles one way, and ended up sleeping in the car at rest areas along I 95 or I 84 on many occasions, but never missed the work on following day. I understand that this was my personal decision and I should not feel bad about compromises if any I had to make neither should I blame others for our circumstances.” Participant Q

Abbreviations: HR, human resources; NC, North Carolina; PTSD, posttraumatic stress disorder.

#### Stress

Psychosocial reactions—including isolation, hopelessness, and intimidation—are unique stressors that, when compounded, create a persistently stressful mental state that may develop into clinical conditions. At least 1 narrator (participant K), albeit a witness, described stress resulting directly from their shared decidedly hostile work environment.

#### Depression and Anxiety

Respondents, such as participants L and M, commonly described how hostile workplaces foster environments that, over time and after repeated exposure to PD, can cause anxiety and depression. Beyond directly causing these issues, workplace environments could also worsen previously existing conditions. For example, participant M shared how their supervisor was increasingly less accommodating of grief and bereavement after the death of participant M’s mother.

#### Posttraumatic Stress Disorder

Long-term harm can result from negative workplace experiences and may require treatment to fully heal individuals experiencing such abuse. PTSD, defined as a psychiatric disorder that can occur in people who have experienced or witnessed a traumatic event,^16^ is a clinical condition that can be treated effectively by a mental health practitioner. In participant O’s witness narrative, a minoritized colleague experienced not just an environment rife with PD that was associated with a host of harmful health outcomes, specifically stress- and/or medication-related weight gain, hair loss, clinical depression, but also PTSD, the last of which was not addressed until after the colleague had found a new job.

#### Changes in Blood Pressure

At least 1 respondent reported increased blood pressure. In participant P’s personal narrative, a supervisor’s toxic behavior was directly tied to decreases in the narrator’s health, specifically weight gain (15 pounds) and increased blood pressure despite a self-reported history of normal blood pressure.

#### Changes in Sleep Behaviors

In 1 of the more extreme cases, participant Q overexerted himself to meet the demands of his new position, including losing sleep to make the long drive to work (≥3 hours), missing key moments in his wife’s pregnancy, and forgoing vacation time. Although participant Q stated that each of these decisions was made of his own volition, the implied consequences of losing his job and, by extension, visa status, are clearly powerful motivating factors call into question the true nature of his voluntary actions.

## Discussion

Within health care organizations, negative group and interpersonal interactions have lasting impacts on how faculty, staff, and students perceive their role in the organizational hierarchy, respond to their colleagues’ actions, and embody salient experiences. In this qualitative study, we identified a range of health outcomes associated with PD and exclusion among employees and trainees of a large urban health care institution. Health care institutions are obliged to prevent discrimination among their workforces and must enact structural changes to protect the health and wellness of their employees, particularly those from historically marginalized populations. These findings on the reported effects of workplace experiences have implications for improving broader institutional workforce and organizational culture by addressing structural factors that contribute to individual employee experiences.

Although some participants attributed feeling excluded to actions by specific individuals and excused the larger institution, work culture is a driving force of individual behaviors and a natural microcosm of societal perspectives, both good and bad, suggesting that increasing inclusion may be most effectively addressed at the organizational level. For example, gone unchecked by bystanders, a racially charged comment made during a faculty meeting can take on added significance, specifically because no one else stood up to the aggressor or expressed their disagreement. On an aggregate level, unchecked comments and lack of bystander intervention are examples of workplace cultural factors that normalize and legitimize harmful interpersonal dynamics, leading to adverse health outcomes, a concept that has previously been defined as structural racism in the context of race,^17^ or structural violence more broadly.^18^ Structural factors such as climate or policies may represent organizational points of intervention, and this indicates we must target units larger than the individual in designing interventions to improve workforce inclusion and wellness and reduce discrimination and exclusion.

Organizational culture and interactions impacted workforce and students’ and trainees’ mental and physical health primarily through stress and anxiety. Many participants prioritized their work over their health. Long term, this trade-off led to changes in sleep, weight, and affect; in extreme cases respondents described changes to blood pressure and even the development of depression symptoms. Social isolation resulting from group-level exclusion was primarily associated with interpersonal group dynamics and power imbalances. For example, employees and/or students and trainees may be more likely to withdraw or be excluded if the primary antagonist is a supervisor or superior. One may falsely presume that certain groups of individuals would be immune from the effects of group power dynamics, in particular senior faculty and administrators; however, we observed that such groups were equally impacted by negative experiences both witnessed and personally experienced.

To comprehensively prevent exclusion and improve workforce wellness, we must consider an organizational-level intervention that will reshape climate, culture, and workplace norms. Examples of effective organizational interventions include work process restructuring and professional development, such as implicit bias training, leadership training, employee assistance programs, bystander intervention training, and team cohesion and professionalism.^19,20,21^ Beyond offering trainings and professional development, health care institutions must monitor health and discrimination among their employees. We identified several health outcomes that were associated with discriminatory workplace experiences, including changes in sleep behavior, depression and anxiety, and even hypertension; these health outcomes should be included in any monitoring, evaluation, or surveillance studies that health care employers may conduct.

Creating diverse and inclusive environments in which to work and learn can play a vital role in reducing perceptions of exclusion and discrimination. For example, higher levels of job satisfaction are a potential moderator of the outcomes of workplace discrimination.^22^ At the same time, employers may also provide employees with resources to reduce the harmful impact of workplace experiences. Workplace interventions have been implemented to address several health-related issues, such as interpersonal violence,^23^ nutrition and physical activity,^24^ sickness absenteeism,^25^ cancer,^26^ and mental health.^27^

On the basis of our findings, we conclude that individual-based workplace interventions (eg, stress reduction) could have the most impact by focusing on employees who have the lowest agency and ability to advocate for their own health, such as staff, junior faculty, women (particularly new or expecting mothers), members of minority racial and ethnic groups, as well as those with intersecting identities. For treatment, an individual-level intervention, organizations must help individuals experiencing discrimination and exclusion address their adverse experiences by mitigating the effects on their health. For example, organizations could promote a culture of self-care among their employees, which includes mindfulness, coping strategies, and stress reduction, or through clinical therapy, which includes psychotherapy, cognitive behavioral therapy, counseling, and medication.^19^ To ensure that employees are able to access self-care options and further demonstrate a commitment to employee health and wellness, institutions should implement cost-free workplace interventions.

By reducing workplace discrimination–related stress and preventing stressors, health care employers not only stand to improve the quality of life for their workers, but also improve the provision of health care services to patients and other health care consumers.^6^ Long-term exposure to workplace stressors, including discrimination and exclusion, can impact employees’ productivity as well as increase stress and anxiety levels, which have been shown to affect physical health through biological mechanisms such as allostasis.^28,29^ Allostasis is part of the body’s homeostatic process where environmental and psychosocial stressors cause physiological changes, such as elevated heart rate, blood pressure, or cortisol levels, in the long term, contributing to increased wear and tear through a chronically high allostatic load.^30,31^ Given the demographic characteristics of our sample and the prevalence of negative workplace experiences, structural racism and other higher-level factors (eg, antidiscrimination policies, recourse options, or the lack thereof) likely play a role in perpetuating a work climate where groups of employees are at higher risk not only for adverse workplace experiences but also for known health sequelae. For example, many of the narratives we analyzed described frustration with the lack of diversity among institutional leadership, increased scrutiny of people of color’s work and job performance, and failed attempts to create change by reporting discrimination through designated channels (eg, human resources, supervisors).

To our knowledge, this study is one of the first to qualitatively assess PD by employees and trainees across an entire health care system, and we found a variety of reported effects of discrimination as well as coping mechanisms to deal with lived experiences. These findings contribute to a well-established body of literature^9,32,33,34,35,36^ showing that women and minority populations share a disproportionate burden of workplace discrimination. In 2020, the US Equal Employment Opportunity Commission reported that among all cases of discrimination, 32.7% were related to race and 31.7% were related to sex.^37^ Our study highlights the need for multifaceted workplace interventions to increase inclusion and wellness through prevention and treatment of adverse workplace experiences, and this applies not only to in-person workplaces but also to virtual work settings.

By forcing many workers into all-virtual work settings, the COVID-19 pandemic may have had a negative impact on organizational efforts to combat workplace discrimination, in particular workplace antidiscrimination and implicit bias programming.^38^ For example, a 2021 survey of US workers found a 19% decrease in employees having received antidiscrimination trainings between February 2020 and February 2021 (57% vs 38%).^39^ On the other hand, 7 in 10 of the employees surveyed believed their company to be committed to diversity, equity, and inclusion.^39^ Given robust evidence that racial and ethnic minority groups have experienced increased discrimination during the COVID-19 pandemic, in particular Black and Asian American individuals,^40^ and an ongoing dialogue about police brutality and the Black Lives Matter movement, antidiscrimination training in the workplace has only increased in relevance and importance.^38^ Future research is needed to understand the true impact of COVID-19 on perceived workplace discrimination.

### Limitations and Strengths

Our study is not without its limitations. The nature of our call-for-narratives campaign likely increased the potential for selection bias, as individuals with salient experiences related to inclusion and diversity efforts may have been more likely to respond and provide details than unaffected individuals. However, our study sample was demographically diverse, skewing toward women and minority racial and ethnic groups, both of which are populations with well-documented histories of discrimination, oppression, and health disparities. Future studies may consider recruiting a sample that better represents the workforce and trainee populations.

## Conclusions

The rich narratives that we analyzed revealed a continuum of negative outcomes on employee health and well-being associated with PD in the workplace. Decreasing workplace discrimination and exclusion may be an effective method for reducing employee and trainee stress and anxiety and related ailments. Interventions to increase inclusion may be most effective at the organizational level, whereas interventions to treat the outcomes of adverse workplace experiences should be directed toward high-risk groups that likely have proportionately more recipients of abuse, such as historically marginalized populations. Ongoing monitoring and evaluation related to organizational inclusion and diversity efforts should include measures of inclusion or lack thereof and associated health outcomes. Looking forward, employers interested in employee well-being must consider the role of exclusion and discrimination in designing health promotion programming, as well as which groups most should receive attention in these efforts.

## References

[1] Sherbin L, Rashid R. Diversity doesn’t stick without inclusion. *Harvard Business Review*. February 1, 2017. Accessed December 17, 2021. https://hbr.org/2017/02/diversity-doesnt-stick-without-inclusion

[2] Carter RT, Johnson VE, Kirkinis K, Roberson K, Muchow C, Galgay C. A meta-analytic review of racial discrimination: relationships to health and culture. Race Soc Probl. 2019;11:15-32. doi:10.1007/s12552-018-9256-y

[3] Chavez LJ, Ornelas IJ, Lyles CR, Williams EC. Racial/ethnic workplace discrimination: association with tobacco and alcohol use. Am J Prev Med. 2015;48(1):42-49. doi:10.1016/j.amepre.2014.08.013 25441232 PMC4274219

[4] Volmer J, Fritsche A. Daily negative work events and employees’ physiological and psychological reactions. Front Psychol. 2016;7:1711. doi:10.3389/fpsyg.2016.01711 27877145 PMC5099156

[5] CareerAddict. Employee turnover: why people quit their jobs. January 2020. Accessed April 19, 2021. https://www.careeraddict.com/CareerAddict-Study-Quit.pdf

[6] Ruotsalainen JH, Verbeek JH, Mariné A, Serra C. Preventing occupational stress in healthcare workers. Cochrane Database Syst Rev. 2014;(11):CD002892. doi:10.1002/14651858.CD002892.pub3 25391582

[7] Krieger N, Waterman PD, Hartman C, . Social hazards on the job: workplace abuse, sexual harassment, and racial discrimination—a study of Black, Latino, and White low-income women and men workers in the United States. Int J Health Serv. 2006;36(1):51-85. doi:10.2190/3EMB-YKRH-EDJ2-0H19 16524165

[8] Dolezsar CM, McGrath JJ, Herzig AJM, Miller SB. Perceived racial discrimination and hypertension: a comprehensive systematic review. Health Psychol. 2014;33(1):20-34. doi:10.1037/a0033718 24417692 PMC5756074

[9] McCord MA, Joseph DL, Dhanani LY, Beus JM. A meta-analysis of sex and race differences in perceived workplace mistreatment. J Appl Psychol. 2018;103(2):137-163. doi:10.1037/apl0000250 29016163

[10] Freimann T, Pääsuke M, Merisalu E. Work-related psychosocial factors and mental health problems associated with musculoskeletal pain in nurses: a cross-sectional study. Pain Res Manag. 2016;2016:9361016. doi:10.1155/2016/9361016 27885319 PMC5112316

[11] Adesoye T, Mangurian C, Choo EK, Girgis C, Sabry-Elnaggar H, Linos E; Physician Moms Group Study Group. Perceived discrimination experienced by physician mothers and desired workplace changes: a cross-sectional survey. JAMA Intern Med. 2017;177(7):1033-1036. doi:10.1001/jamainternmed.2017.1394 28492824 PMC5818808

[12] Peterson U, Demerouti E, Bergström G, Samuelsson M, Åsberg M, Nygren A. Burnout and physical and mental health among Swedish healthcare workers. J Adv Nurs. 2008;62(1):84-95. doi:10.1111/j.1365-2648.2007.04580.x 18352967

[13] Aysola J, Barg FK, Martinez AB, . Perceptions of factors associated with inclusive work and learning environments in health care organizations: a qualitative narrative analysis. JAMA Netw Open. 2018;1(4):e181003. doi:10.1001/jamanetworkopen.2018.1003 30646094 PMC6324264

[14] Hacer TY, Ali A. Burnout in physicians who are exposed to workplace violence. J Forensic Leg Med. 2020;69:101874. doi:10.1016/j.jflm.2019.101874 31669822

[15] Weisberg J, Sagie A. Teachers’ physical, mental, and emotional burnout: impact on intention to quit. J Psychol. 1999;133(3):333-339. doi:10.1080/00223989909599746

[16] American Psychiatric Association. Diagnostic and Statistical Manual of Mental Disorders. 5th ed. American Psychiatric Association; 2013.

[17] McCluney CL, Schmitz LL, Hicken MT, Sonnega A. Structural racism in the workplace: does perception matter for health inequalities? Soc Sci Med. 2018;199:106-114. doi:10.1016/j.socscimed.2017.05.039 28552294 PMC5696122

[18] Farmer PE, Nizeye B, Stulac S, Keshavjee S. Structural violence and clinical medicine. PLoS Med. 2006;3(10):e449. doi:10.1371/journal.pmed.0030449 17076568 PMC1621099

[19] Awa WL, Plaumann M, Walter U. Burnout prevention: a review of intervention programs. Patient Educ Couns. 2010;78(2):184-190. doi:10.1016/j.pec.2009.04.008 19467822

[20] Frye V, Camacho-Rivera M, Salas-Ramirez K, . Professionalism: the wrong tool to solve the right problem? Acad Med. 2020;95(6):860-863. doi:10.1097/ACM.0000000000003266 32134778

[21] Powers BW, White AA, Oriol NE, Jain SH. Race-conscious professionalism and African American representation in academic medicine. Acad Med. 2016;91(7):913-915. doi:10.1097/ACM.0000000000001074 26760060

[22] Di Marco D, López-Cabrera R, Arenas A, Giorgi G, Arcangeli G, Mucci N. Approaching the discriminatory work environment as stressor: the protective role of job satisfaction on health. Front Psychol. 2016;7:1313. doi:10.3389/fpsyg.2016.01313 27625625 PMC5003878

[23] Runyan CW, Zakocs RC, Zwerling C. Administrative and behavioral interventions for workplace violence prevention. Am J Prev Med. 2000;18(4)(suppl):116-127. doi:10.1016/S0749-3797(00)00147-1 10793287

[24] Maes L, Van Cauwenberghe E, Van Lippevelde W, . Effectiveness of workplace interventions in Europe promoting healthy eating: a systematic review. Eur J Public Health. 2012;22(5):677-683. doi:10.1093/eurpub/ckr098 21785115

[25] Odeen M, Magnussen LH, Maeland S, Larun L, Eriksen HR, Tveito TH. Systematic review of active workplace interventions to reduce sickness absence. Occup Med (Lond). 2013;63(1):7-16. doi:10.1093/occmed/kqs198 23223750 PMC3537115

[26] Sorensen G, Thompson B, Glanz K, . Work site-based cancer prevention: primary results from the Working Well Trial. Am J Public Health. 1996;86(7):939-947. doi:10.2105/AJPH.86.7.939 8669517 PMC1380434

[27] Czabała C, Charzyńska K, Mroziak B. Psychosocial interventions in workplace mental health promotion: an overview. Health Promot Int. 2011;26(1)(suppl):i70-i84. doi:10.1093/heapro/dar050 22079937

[28] Logan JG, Barksdale DJ. Allostasis and allostatic load: expanding the discourse on stress and cardiovascular disease. J Clin Nurs. 2008;17(7B):201-208. doi:10.1111/j.1365-2702.2008.02347.x 18578796

[29] Krieger N, Sidney S. Racial discrimination and blood pressure: the CARDIA Study of young black and white adults. Am J Public Health. 1996;86(10):1370-1378. doi:10.2105/AJPH.86.10.1370 8876504 PMC1380646

[30] McEwen BS. Stress, adaptation, and disease: allostasis and allostatic load. Ann N Y Acad Sci. 1998;840:33-44. doi:10.1111/j.1749-6632.1998.tb09546.x 9629234

[31] McEwen BS, Seeman T. Protective and damaging effects of mediators of stress: elaborating and testing the concepts of allostasis and allostatic load. Ann N Y Acad Sci. 1999;896:30-47. doi:10.1111/j.1749-6632.1999.tb08103.x 10681886

[32] Krieger N. Racial and gender discrimination: risk factors for high blood pressure? Soc Sci Med. 1990;30(12):1273-1281. doi:10.1016/0277-9536(90)90307-E 2367873

[33] Bendick M, Jackson CW, Reinoso VA. Measuring employment discrimination through controlled experiments. Rev Black Polit Econ. 1994;23(1):25-48. doi:10.1007/BF02895739

[34] Pavalko EK, Mossakowski KN, Hamilton VJ. Does perceived discrimination affect health? longitudinal relationships between work discrimination and women’s physical and emotional health. J Health Soc Behav. 2003;44(1):18-33. doi:10.2307/1519813 12751308

[35] Randall AK, Totenhagen CJ, Walsh KJ, Adams C, Tao C. Coping with workplace minority stress: associations between dyadic coping and anxiety among women in same-sex relationships. J Lesbian Stud. 2017;21(1):70-87. doi:10.1080/10894160.2016.1142353 27611568

[36] Zurbrügg L, Miner KN. Gender, sexual orientation, and workplace incivility: who is most targeted and who is most harmed? Front Psychol. 2016;7:565. doi:10.3389/fpsyg.2016.00565 27199804 PMC4851979

[37] US Equal Employment Opportunity Commission. Charge statistics (charges filed with EEOC) FY 1997 through FY 2020. Accessed October 5, 2021. https://www.eeoc.gov/eeoc/statistics/enforcement/charges.cfm

[38] Doherty K. COVID-19 was bad for discrimination training. Association for Talent Development. April 15, 2021. Accessed April 19, 2021. https://www.td.org/atd-blog/covid-19-was-bad-for-discrimination-training

[39] True Global Intelligence, Vyond Team. #VyondTheSurface: what COVID-19 changed in a year. FleishmanHilliard. Accessed November 22, 2021. https://view.ceros.com/vyond/vyondthesurface-landing-page-2-1-1

[40] Ruiz NG, Horowitz J, Tamir C. Many Black and Asian Americans say they have experienced discrimination amid the COVID-19 outbreak. Pew Reseearch Center. July 1, 2020. Accessed December 17, 2021. https://www.pewresearch.org/social-trends/2020/07/01/many-black-and-asian-americans-say-they-have-experienced-discrimination-amid-the-covid-19-outbreak/

